# 1.5 T MR-Guided Daily Adapted SBRT on Lymph Node Oligometastases from Prostate Cancer

**DOI:** 10.3390/jcm11226658

**Published:** 2022-11-10

**Authors:** Luca Nicosia, Giovanna Trapani, Michele Rigo, Niccolò Giaj-Levra, Rosario Mazzola, Edoardo Pastorello, Francesco Ricchetti, Francesco Cuccia, Vanessa Figlia, Matilde Fiorini, Filippo Alongi

**Affiliations:** 1 Advanced Radiation Oncology Department, IRCCS Sacro Cuore Don Calabria Hospital, Cancer Care Center, 37024 Negrar di Valpolicella, Italy; 2 Clinical Research Unity, IRCCS Sacro Cuore Don Calabria Hospital, Cancer Care Center, 37024 Negrar di Valpolicella, Italy; 3 University of Brescia, 25121 Brescia, Italy

**Keywords:** adaptive radiotherapy, lymph node, MR-linac, MRgRT, oligometastases, SBRT, prostate cancer

## Abstract

**Introduction:** The aim of our study was to evaluate the efficacy and toxicity of a daily adaptive MR-guided SBRT on 1.5 T MR-linac in patients affected by lymph node oligometastases from PCa. **Materials and Methods:** The present study is a prospective observational study conducted in a single institution (protocol n°: MRI/LINAC n. 23748). Patients with oligometastatic lymph nodes from PCa treated with daily adaptive MR-guided SBRT on 1.5 T MR-linac were included in the study. There was a minimum required follow-up of 3 months after SBRT. The primary end-point was local progression-free survival (LPFS). The secondary end-points were: nodal progression-free survival (NPFS), progression-free survival (PFS), and toxicity. **Results:** A total of 118 lymph node oligometastases from PCa were treated with daily adaptive 1.5 T MR-guided SBRT in 63 oligometastatic patients. Of the patients, 63.5% were oligorecurrent and 36.5% were oligoprogressive. The two-year LPFS was 90.7%. The median NPFS was 22.3 months and the 2-year NPFS was 46.5%. Receiving hormone therapy before SBRT was correlated with a lower NPFS at the multivariate analysis (1 y NPFS 87.1% versus 42.8%; *p* = 0.002–HR 0.199, 95% CI 0.073–0.549). Furthermore, the oligorecurrent state during ADT was correlated with a lower NPFS than was the oligoprogressive state. The median PFS was 10.3 months and the 2-year PFS was 32.4%. Patients treated with hormone therapy before SBRT had a significantly lower 1-year PFS the others (28% versus 70.4%; *p* = 0.01–HR 0.259, 95% CI 0.117–0.574). No acute and late toxicities occurred during treatment. **Conclusions:** The present study is the largest prospective study of 1.5 T lymph node SBRT on MR-linac in patients with PCa. Lymph node SBRT by 1.5 T MR-linac provides high local control rates with an excellent toxicity profile.

## 1. Introduction

Prostate cancer (PCa) is the second most frequent cancer and the fifth leading cause of cancer death in men worldwide [1]. PCa incidence and mortality rates are closely related to the widespread use of PSA screening as it allows for early cancer detection, but it also increases the identification of latent prostate cancer. Furthermore, advances in imaging techniques in recent years have led to an increase in the detection of metastatic and oligometastatic disease and thus to a growing interest in metastasis-directed therapies (MDT) [2]. Several prospective studies have confirmed a disease progression and survival improvement in oligometastatic patients when MDT is implemented in the therapeutic workflow [3,4,5]. Based on international guidelines, the current standard of treatment for metastatic PCa is still androgen deprivation therapy (ADT), which is eventually associated with other systemic therapies, with no specific indications for the subgroup of oligometastatic patients [6]. The ideal time to initiate ADT (immediate or delayed until symptoms occur) remains controversial [7]. In recent years, growing evidence has suggested that local treatment of the oligometastatic PCa could be a therapeutic option due to the high local control level and good tolerability, aiming to delay the initiation of systemic treatments [4]. Recently, the Advanced Prostate Cancer Consensus Conference recommended the association of ADT with local treatment to all oligorecurrent lesions, which was also due to a better toxicity profile of the MDT versus chemotherapy or ADT [7,8]. Lymph node metastases are a particular site of PCa oligometastatic disease amenable to MDT and might identify a less severe degree of metastatic PCa [9]. Several studies, both prospective and retrospective, have evaluated the role of stereotactic body radiotherapy (SBRT) in the management of lymph node oligometastases, reporting few side effects and adequate local control levels [10,11,12]. The widespread use of metabolic imaging in the very early phase of the PCa metastatic disease has permitted the early identification of metastatic lesions, also in millimeters [13]. Despite image-guided radiotherapy significantly increasing treatment accuracy, there might be some dosimetric and imaging limitations when lesions are small or close to organs at risk or to vessels [14,15]. Moreover, standard radiotherapy on conventional linac does not allow clinicians to modify treatment plans in the case of lesion displacement or when organs at risk (OARs) (i.e., bowel loops) fall within the treatment field, as may occur for abdominal targets. The recent introduction of MR-linac might overcome this problem by allowing for daily treatment adaptation and exploiting the high tissue contrast provided by MR images [16]. Recent evidence has shown dosimetric improvements to bowel loops in lymph node metastases treated with SBRT on a 1.5 T MR-linac [14]. However, to date, clinical data are limited by the small retrospective series and short follow-up periods [17,18]; therefore, little is known regarding the clinical impact of MR-guided radiotherapy (MRgRT). The aim of the present prospective study is to evaluate the efficacy and safety of MR-guided SBRT for lymph node oligometastases in patients affected by PCa.

## 2. Material and Methods

The present study is a prospective observational study conducted at the IRCSS Sacro Cuore Don Calabria (Negrar di Valpolicella, Verona, Italy), approved in April 2019 by the local Ethics Committee (MRI/LINAC n. 23748). Patients with metastatic lymph nodes from PCa treated with daily adaptive MR-guided SBRT on 1.5 T MR-linac were included in the study. The inclusion criteria were:

- age ≥ 18 years.
- histological diagnosis of PCa
- performance status ECOG (Eastern Cooperative Oncology Group Criteria) ≤ 2
- oligometastatic or oligoprogressive disease of the lymph nodes (≤5 nodal disease sites) from PCa diagnosed with PET-choline or PET-PSMA
- primary tumor controlled (prostate cancer locally treated with surgery/RT/other or not in radiological progression at the time of SBRT)
- a minimum follow-up period of 3 months after SBRT

The exclusion criteria were:

- general contraindications for 1.5 T MR
- claustrophobia

Patients could have received ADT before or during SBRT. ADT before SBRT was administered in de novo oligometastatic patients and oligorecurrent disease. Initial treatments included radical surgery, radical radiotherapy, HIFU, or hormone therapy (the latter in the case of de novo metastatic disease).

### 2.1. Treatment Procedure

The pre-treatment imaging consisted of a planning CT (Somatom AS, Siemens, Germany), with a slice thickness of 3 mm, and a 3D T2-weighted MRI (T2w) (1.5 T Philips Ingenia) (1 mm slice thickness). The same MR scans were performed daily for the treatment procedures. All scans were acquired with patients in the supine position, with a head-first orientation, with support for the knees and arms on the chest in the case of pelvic or lower abdominal targets. An anterior coil was placed on the patient’s body to maximize the signal-to-noise ratio. The same patient and coil position were reproduced for each treatment fraction.

Planning CT and MR images were rigidly co-registered based on bone anatomy, primarily to obtain bulk densities for each tissue. Target lymph node volume (GTV) and OARs were contoured on an MRI with the aid of co-registered CT and staging diagnostic exams (i.e., PET). Therefore, GTV was delineated on MR as the entire visible tumor and was considered equal to the clinical target volume (CTV). The OARs were configured as avoidance structures, depending on the proximity to the target. All volumes, including OARs, were delineated by a Radiation Oncologist experienced in MR imaging, according to the guidelines, and specifically contouring the portion of the bowel close to the target with a craniocaudal extension up to 1 cm above and below.

The GTV to planning target volume (PTV) margin was created by adding 2 to 4 mm, based on the distance and movement between the target and OARs. More specifically, the strategy for defining the PTV comprised a progressive margin reduction from 4 to 2 mm within the protocol.

The pretreatment plan was generated using the Monaco planning system 5.40.01 (Elekta AB, Stockholm, Sweden). Offline intensity-modulated radiotherapy (IMRT) plans were optimized on MR scanning, typically with 10–11 fixed beam angles. A flattening filter-free (FFF) photon beam was employed.

Treatment was usually prescribed to ensure that at least 95% of the PTV received at least 95% of the prescribed dose. The maximum dose in the PTV was not to exceed 107%. Lower PTV coverage was only accepted to comply with maximum dose constraints for surrounding OARs. All pretreatment plans passed the standard quality control procedure before the first treatment session.

The daily workflow consisted of an initial T2w 3D MR scan, rigidly fused with the pretreatment MR scan. The contours of the target and the OARs were automatically propagated to the scan of the day from a deformable registration and used for the optimization of the plan. In all sessions, the treatment was delivered using the adaptive workflow, choosing between two possibilities: adapt to position (ATP) or adapt to shape (ATS) [18,19]. The ATS approach is the most robust because it allows a complete adaptation of the contours (manually or by deformable registration) and a full re-planning, considering any movement or change in the shape or volume of the OARs. A possible limitation of ATP, in which only the isocenter is modified, is that the exact dose received by the OARs in each fraction is unknown, as the same contours are used as an avoidance framework for re-planning and the outlined OARs do not represent the OARs acquired during the MRI performed daily. Consequently, although the ATS approach might require more time, it allows for a better assessment of the real dose received by the organs close to the target. Therefore, the choice of the ATP approach was usually adopted to speed up this procedure only in the case of no substantial differences in the daily anatomy compared to the reference imaging. In the meantime, a complete re-optimization of the plan was performed, and a verification MR before treatment administration was acquired to evaluate any target and OARs movement [19]. Afterward, the Radiation Oncologist and the Physicist evaluated the new treatment plan and verified the absence of important movements through the visual comparison (iso-iso) of the images and contours. If a major change in the anatomy of the OARs or the target was found, a new ATP or ATS was started; otherwise, the radiation treatment was administered. Before and during RT administration, intra-fraction motion was monitored with a real-time 2D cine-MRI (T2/T1-weighted balanced free precession at steady state) acquired in the coronal and sagittal views. Finally, another MR scan was acquired after treatment for offline recalculation purposes, such as the assessment of intrafraction target coverage. The entire session time, defined as the time between patient entry and exit from the treatment room, was measured by a radiotherapy technician (RTT). Treatment duration for lymph node SBRT on an MR-linac was between 22 and 31 min (average of 25 min).

### 2.2. Endpoints, Follow-Up, and Statistics

The primary endpoint was local progression-free survival (LPFS). The secondary endpoints were acute and late toxicity, nodal progression-free survival (NPFS), and disease progression-free survival (PFS). LPFS was defined as the time between the onset of SBRT and the radiological diagnosis of local recurrence. NPFS was defined as the time between SBRT and the radiological diagnosis of nodal progression. PFS was defined as the time between SBRT and disease progression (both biochemical and radiological). Acute and late toxicity were defined according to the Common Terminology Criteria for Adverse Events (CTCAE) v.5.0 scale. Toxicity was prospectively recorded in a specific form by the treating physician or nurse before treatment and during follow-up (3 and 6 months after SBRT administration and every 6 months for two years afterward).

The oligometastatic state was defined according to the ESTRO-ASTRO Consensus [20]. Oligoprogressive disease was characterized by disease progression in ≤3 sites while on systemic therapy. An oligorecurrence state during disease at least three months after the initial diagnosis (‘metachronous’) was defined as a state of metachronous limited recurrence.

Follow-up was performed with PSA every 3 months for the first 2 years after SBRT, and every 6 months afterward. Response to treatment and radiological staging with choline/PSMA PET-TC was performed in the case of a PSA rise after SBRT.

Univariate analyses were performed with the Kaplan–Meyer method. The log-rank test was used to determine the difference between the corresponding curves. The following independent variables were evaluated for LPFS, NPFS, and PFS: biological effective dose (BED) Gy 1.5, mm of PTV expansion, median PSA value before SBRT, Gleason score at diagnosis, ADT administration before or during SBRT, oligometastatic state (oligorecurrent versus oligoprogressive), and lymph node site (pelvis versus extrapelvic). The multivariate analysis was performed with the Cox regression model; all the clinically relevant variables in the univariate analysis (*p* < 0.2) were included in the analysis. The BED was calculated using an alpha/beta ratio of 1.5 Gy. The statistical analysis was performed using the SPSS v.20.0 software (IBM software, Armonk, NY, USA). A *p*-value < 0.05 indicated a significant correlation.

## 3. Results

Between November 2019 and March 2022, 118 metastatic lymph nodes from PCa were treated with daily adaptive 1.5 T MR-guided SBRT in 63 oligometastatic patients. The median age was 71 years (range 54–87). Treatments for the primary tumors were surgery (81%), RT (9.4%), HIFU (3.2%), and ADT (6.4%). The median PSA before SBRT was 2.23 ng/mL. Regarding the oligometastatic state, 63.5% of patients were oligorecurrent and 36.5% were oligoprogressive. The patients’ characteristics are summarized in Table 1. The median diameter of the lymph node metastases was 6 mm (range 2–26). Of the treated lymph nodes, 74.6% were located in the pelvis and 25.4% were extrapelvic. The median BED was 198.33 Gy 1.5 (range 65–315). The lesion and treatment characteristics are summarized in Table 2.

### 3.1. Local Control, Survival, and Toxicity

**Figure 1:**
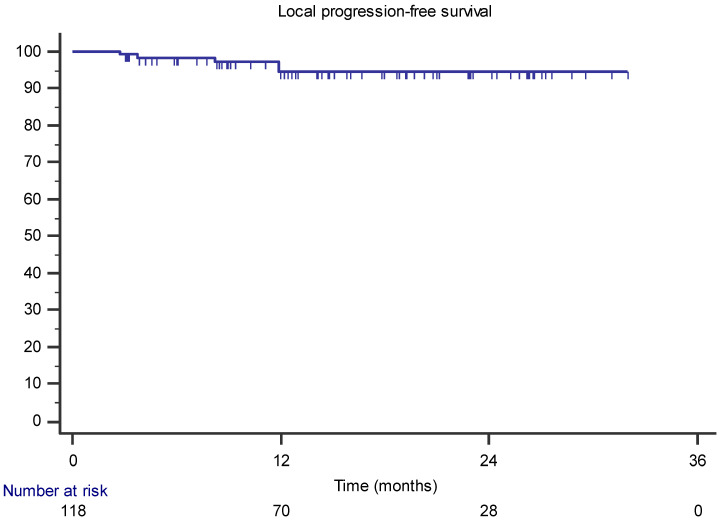
Kaplan–Meier curve showing the local progression-free survival.

The median follow-up period was 17 months (range 3–34). At the last follow-up, only one patient had died from disease progression. The 1- and 2-year LPFSs were 94.5% and 90.7%, respectively (Figure 1). In univariate analysis, only concomitant hormone therapy and pelvic location of the lymph nodes were correlated with improved LPFS. However, no factor remained significantly associated in the multivariate analysis. The following isotropic PTV expansions were used for the MR-guided lymph node SBRT: 36 lesions (30.5%) of 4 mm, 46 lesions (39%) of 3 mm, and 36 (30.5%) lesions of 2 mm; however, there was no statistically significant difference in LPFS between the three groups (*p* = 0.42).

**Figure 2:**
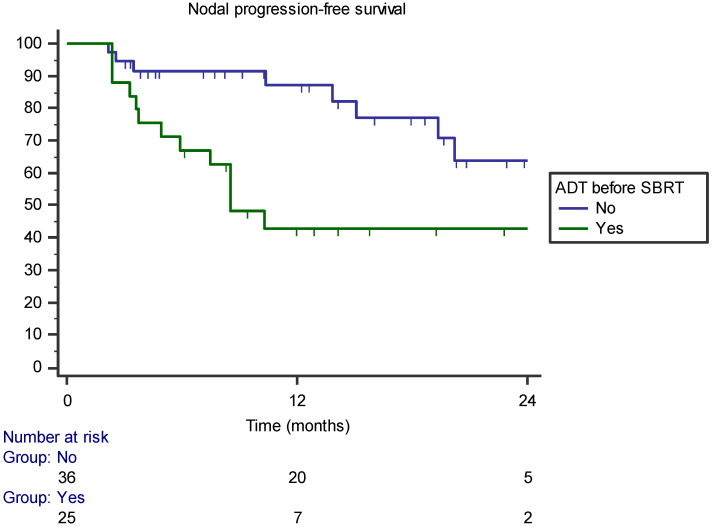
Kaplan–Meier curve showing nodal progression-free survival stratified by androgen deprivation therapy (ADT) before SBRT administration.

**Figure 3:**
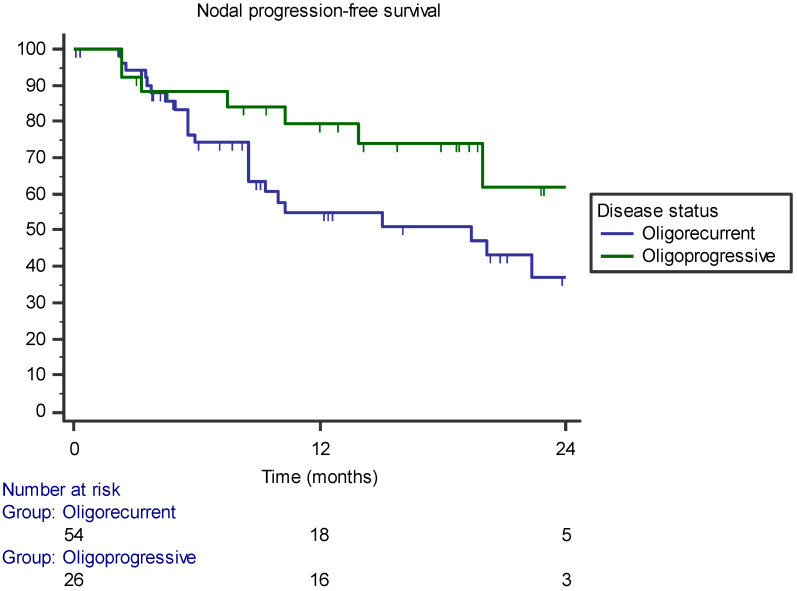
Kaplan–Meier curve showing nodal progression-free survival stratified by oligometastatic state (oligorecurrent versus oligoprogressive).

The median NPFS was 22.3 months (range 16–28 months) and the 1- and 2-year NPFSs ere 65.4% and 46.5%, respectively. In the univariate analysis, receiving hormone therapy before SBRT was correlated with a lower NPFS (*p* = 0.014) (Table 3). The factor remained significantly correlated in the multivariate analysis. In particular, the 1-year NPFS was 87.1% for patients not previously treated with ADT and 42.8% for those previously treated with ADT (*p* = 0.002–HR 0.199, 95% CI 0.073–0.549; Figure 2). Furthermore, the oligorecurrent state during ADT was correlated with a lower NPFS than was the oligoprogressive state. More specifically, the 1-year NPFSs were 79.4% and 54.7% for the oligoprogressive and oligorecurrent state, respectively (*p* = 0.018–HR 3.679, 95% CI 1.247–10.851; Figure 3). The multivariate analysis is shown in Table 4.

**Figure 4:**
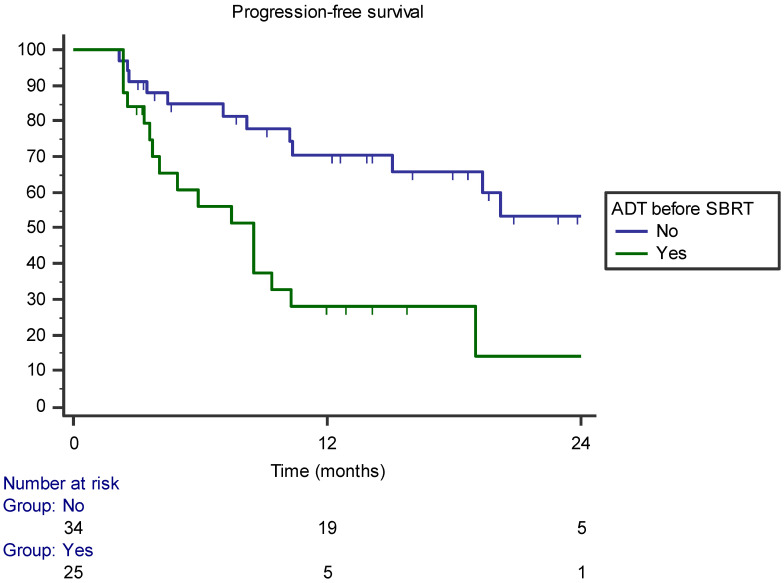
Kaplan–Meier curve showing progression-free survival stratified by androgen deprivation therapy (ADT) before SBRT administration.

The median PFS was 10.3 months (range 5–20.5) and the 1- and 2-year PFSs were 49.8% and 32.4%, respectively. In the univariate analysis, hormone therapy before SBRT was associated with a worse PFS (*p* = 0.002). In multivariate analysis, the covariate remained significantly associated with PFS. In particular, patients treated with hormone therapy before SBRT had a significantly lower 1-year PFS than patients not treated with hormone therapy (28% versus 70.4%, respectively) (*p* = 0.01–HR 0.259, 95% CI 0.117–0.574; Figure 4). No acute and late toxicities occurred during treatment; all patients completed the treatment.

### 3.2. Pattern of Recurrence

After SBRT, 19 lymph node recurrences occurred, of which 3 (15.8%) were in the same lymph node station, 14 (73.7%) were in a different lymph node site, and 2 (10.5%) were in both. Disease recurrence globally occurred in 47.6% of the patients. The pattern of recurrence was: 50% lymph node only, 23.4% bone only, 13.3% both lymph node and bone, 6.7% biochemical only, 3.3% visceral, and 3.3% in prostate bed (Table 5).

## 4. Discussion

In recent years, several studies have been published on the role of SBRT in the treatment of lymph node oligometastases from PCs. In fact, lymph node oligometastases seem to identify an initial phase in the PCa disease progression characterized by a better prognosis than that of bone or visceral lesions [9].

In the context of oligometastatic lymph node disease, MDT use has resulted in an excellent rate of local control and PFS, without unexpected side effects [4,5,21]. In particular, SBRT is based on the possibility of delivering high radiation doses to small volumes with high precision, generally using cone-beam CT-based methods for image guidance. The consequent greater precision in treatment administration has led to a significant reduction in the intrafraction variability due to set-up errors and physiological changes in the healthy structure position and volume. However, especially in the abdominal-pelvic area, target identification may not always be optimal with cbCT IGRT methods due to lower resolution than conventional CT and low soft tissue contrast [14,15].

In this scenario, the recent introduction of MR-linac technology into clinical practice represents a potential paradigm shift in the implementation of SBRT. Based on a higher anatomical visualization, MR-linac could improve not only the definition of target volumes and OARs due to the image quality of MR, but it may also improve treatment safety and accuracy due to the daily adaptive modality [16]. The latter is a crucial issue, especially in the case of targets close to healthy structures, which are more influenced by daily anatomical variations, such as bowel loops [14,18,22]. The aim of our study was to evaluate the efficacy and toxicity of a daily adaptive MR-guided SBRT on 1.5 T MR-linac in patients affected by lymph node oligometastases from PCa.

To our knowledge, the present study is the largest prospective study of 1.5 T lymph node SBRT on MR-linac in patients with PCa. Previous evidence, mainly retrospective, on the use of SBRT in PCa lymph node oligometastases with conventional linacs has reported local control rates of approximately 93% at two years [10,11,12]. In our experience, in line with these previous experiences, the 2-year local control rate was 94.5%. Specifically, patients were treated with a median total dose of 35 Gy with a median BED of 198.33 Gy 1.5. Most of our patients were treated using the ATS modality (87%), which allows for a more robust calculation of the treatment plan than ATP with a variation in planning time between the two modalities of approximately 2–3 min. In our clinical experience, the increase in planning time should not have reduced the accuracy of the treatment, which is guaranteed by the pre-treatment MR and online verification with the cine-MRI. In a previous study on PCa patients treated with prostate SBRT on MR-linac, it was shown that the longer pre-treatment time (imaging acquisition, contouring, planning, and pre-administration MR verification) did not affect treatment accuracy and dose constraints to OARs were respected when compared with conventional linac [19,22]. The accuracy of the MR-linac allowed us to test the effect of the reduction of the PTV margins on local control compared to the standard of 5 mm on conventional linac. Specifically, we have progressively reduced the margins from 4 to 2 mm, reporting no difference in terms of local control. In a previous study by Jereczek-Fossa et al., patients with metastases from solid tumors (16 of which were on lymph nodes), were treated on Cyberknife with SBRT by applying a margin of 1–2 mm through the implantation of fiducial markers [23]. At a median follow-up of 16 months, there were no local recurrences in the subgroup of lymph node metastases. Although encouraging—but limited to a small population—these results required an invasive procedure. On the contrary, a treatment on MR-linac could guarantee comparable results in terms of local control, without invasive procedures. The reduction of the treatment margin can have important clinical consequences. The main and most direct consequence is the reduction of the dose to the OARs, and further consequences could include the possibility of safely escalating the treatment dose. This approach could be especially useful in patients with cancers different from prostate cancers, with an alpha/beta ratio of greater than 1.5 Gy, in which the advantages of hypofractionation are minor and a higher radiation dose is required to obtain a higher BED [24,25]. The use of smaller treatment volumes could also facilitate a re-challenge with SBRT in patients with recurrence of the disease in the cranial or caudal area to the previous treatment field, limiting possible overlaps with previous treatment fields. This pattern of relapse is, for example, relatively common in PCa, in which up to 50% of patients with lymph node disease may have a cranial recurrence in the same lymph node station previously treated with SBRT [9,10].

The standard of treatment in the case of metastatic prostatic disease is ADT, possibly associated with chemotherapy or second-generation antiandrogens [26,27,28]. However, in the oligometastatic setting, the SABR-COMET study demonstrated that the addition of SBRT to the standard of treatment can increase overall survival [3]. In particular, this phase II study closed early due to the superiority of the experimental arm, which included 99 patients affected by different histologies, including the prostate. The latest update confirmed a median survival advantage of the experimental arm (42.3 versus 17.7 months). In recent years, however, some studies have shown that oligometastatic PCa patients treated with SBRT alone could have a relatively long disease-free interval, thus underlining the need to identify patients who can really benefit from an early onset of ADT or not [11].

For example, in an Italian multicenter study [11], 141 patients treated with SBRT to 209 lesions reported a 2-year ADT-free survival of 47.3%. In a phase II study by Ost et al. [4], patients with oligometastatic prostate cancer underwent SBRT on active disease sites or observation. The results reported increased ADT-free survival of 21 versus 14 months in the experimental and observational arms, respectively. In our study, the 1-year PFS was 49.8%, in line with these previous studies. There is currently no robust evidence on the timing of onset and duration of ADT concomitant with SBRT, as highlighted by a recent review [12] which analyzed 15 studies of SBRT in oligometastatic PCa patients. The authors reported a high heterogeneity in ADT administration concomitant with SBRT in a range between 33% and 100%, and durations between 14.5 and 17.5 months. The study confirmed the high rate of local control and PFS at 2 years after lymph node SBRT of 84% and 38.6%, respectively.

The review also reported generally low rates of acute toxicity after SBRT. Specifically, acute and late G1–G2 toxicity rates were approximately 1.9% and, on average, severe acute and late toxicities ≥G3 occurred in approximately 0.6% of cases [12]. This data is in line with our prospective series in which no acute and late toxicity episodes occurred.

Considering the peculiar pattern of PCa lymph node recurrence after SBRT [9,10], some studies have investigated the role of pelvic irradiation. Lepinoy et al. evaluated the use of whole-pelvis radiotherapy (WPRT) compared to the MDT approach, reporting 3-year PFSs of 88% and 55% for WPRT and MDT, respectively [29]. A recent DEGRO expert group report on PCa [30] recommended treating pelvic-only oligorecurrent lymph node metastases from PCa with WPRT plus a boost to the involved lymph nodes and to consider SBRT alone in nodal extra-pelvic oligorecurrent cases. However, they pointed out that in some situations with low-risk characteristics, such as a PSA doubling time of >10 months and a relapse-free interval from primary curative treatment of >2 years, focal treatment might be considered.

The present study is not without limitations: the use of two different PET tracers (choline and PSMA) in the staging phase which, although they had no impact on local control, the primary end-point of the study, they could limit the analysis of PFS; the absence of stratification by the initial stage of disease; and the lack of a comparison group treated on conventional linac. A comparative analysis in this regard is currently ongoing at our Center. Strengths of the study included the prospective nature and the sample size that, to our knowledge, represents the largest study of lymph node SBRT from PCa treated with 1.5 T MR-linac.

## 5. Conclusions

Lymph node SBRT by 1.5 T MR-linac provides high local control rates with an excellent toxicity profile. This method would to be not inferior to the standard of treatment on conventional linac, although a direct comparison study will be required to confirm this hypothesis. SBRT is also confirmed as a valid therapeutic tool for postponing the initiation of systemic treatment in selected patients. The ability to administer SBRT with the same accuracy, reducing treatment margins, could allow for radiation dose-escalation in order to maximize local control while controlling toxicity. A longer follow-up is necessary to assess the long-term conclusion of this work.

## Figures and Tables

**Table 1:** Patients’ characteristics (*n* = 63).

**Mean age (years) (range)**	71 (54–87)
**ECOG PS**	
• 0	62
• 1	1
**Initial treatment**	
• RP +/− ADT	51 (81%)
• RT +/− ADT	6 (9.4%)
• HIFU/other	2 (3.2%)
• ADT	4 (6.4%)
**Initial stage**	
• T2	17 (27%)
• T3	32 (51%)
• T4	1 (1.5%)
• Unknown	13 (20.5%)
**Initial PSA (ng/mL) (range)**	19.1 (3.5–140)
**Initial Gleason**	
• 6	9 (14%)
• 7	22 (35%)
• >8	28 (44.5%)
• Unknown	4 (6.5%)
**PSA before SBRT (ng/mL) (range)**	2.23 (0.22–36.84)
**Oligometastatic status**	
• Oligoreccurent	40 (63.5%)
• Oligoprogressive	23 (36.5%)
**Imaging pre-SBRT**	
• Choline-PET	12 (19%)
• PSMA-PET	51 (81%)
**N° of treated lesions (per patient)**	
• 1	41 (65%)
• 2	13 (20.5%)
• 3	7 (11.5%)
• 4	2 (3%)
**Total SBRT course**	
• 1	55 (87%)
• 2	7 (11.5%)
• 3	1 (1.5%)

ECOG PS: Eastern Cooperative Oncology Group—Performance Status; RP: radical prostatectomy; ADT: androgen deprivation therapy; RT: radiotherapy; HIFU: high-intensity focused ultrasound; SBRT: stereotactic body radiotherapy; PSMA: prostate specific membrane antigen; PET: positron emission tomography.

**Table 2:** Lesions and treatment characteristics (*n* = 118).

Lesion diameter (mm) (total = 118)	6
Range (mm)	2–26
Lymph node site	
Pelvic	88 (74.5%)
Extrapelvic	30 (25.5%)
SBRT regimen	
Median dose (range)	35 (15–40)
Median dose/fractions (range)	7 (5–21)
Median BED (α/β = 1.5)	198.33 (65–315)

SBRT: stereotactic body radiotherapy; BED: biological effective dose.

**Table 3:** Univariate analysis.

Covariates	LPFS	NPFS	PFS
	*p*		
BED 198.33	0.344	0.968	0.08
PTV margin	0.427	0.496	0.674
PSA 0.82	0.573	0.604	0.494
PSA 0.7	0.17	0.783	0.379
PSA 0.9	0.573	0.604	0.494
Gleason	0.267	0.608	0.795
ADT concomitant	0.07	-	-
ADT before SBRT	0.171	*0.014*	*0.002*
Oligorecurrent versus oligoprogressive	-	0.068	0.158
Pelvic versus extrapelvic	*0.004*	0.076	0.233

LPFS: local progression-free survival; NPFS: nodal progression-free survival; PFS: progression-free survival; BED: biological effective dose; PTV: planning treatment volume; PSA: prostate-specific antigen; ADT: androgen deprivation therapy; SBRT: stereotactic body radiotherapy. Italicized values indicate a significant correlation.

**Table 4:** Multivariate analysis.

Covariates	LPFS	NPFS	PFS
PSA before SBRT < 0.7	*p* = 0.149 (HR 0.134, 95% CI 0.009–2.060)	-	-
Concomitant ADT	*p* = 0.872 (HR 0.805, 95% CI 0–1.705)	-	-
ADT before SBRT	*p* = 0.503 (HR 2.665, 95% CI 0.151–46.966)	*p* = 0.002 (HR 0.199, 95% CI 0.073–0.549)	*p* = 0.01 (HR 0.259, 95% CI 0.117–0.574)
Pelvic versus extrapelvic lymph node	*p* = 0.923 (HR 0, 95% CI 0–8.826)	*p* = 0.51 (HR 1.492, 95% CI 0.454–4.895)	
Oligorecurrent versus oligoprogressive	-	*p* = 0.018 (HR 3.679, 95% CI 1.247–10.851)	*p* = 0.106 (HR 1.975, 95% CI 0.865–4.511)
BED	-	-	*p* = 0.442 (HR 1.784, 95% CI 0.408–7.811)

LPFS: local progression-free survival; NPFS: nodal progression-free survival; PFS: progression-free survival; PSA: prostate-specific antigen; SBRT: stereotactic body radiotherapy; ADT: androgen deprivation therapy; BED: biological effective dose.

**Table 5:** Pattern of recurrence.

Nodal recurrence	
Some nodal level	6 (31.5)
Different nodal level	11 (58)
Both	2 (10.5)
Global disease recurrence	
Nodal	15 (50)
Bone	7 (23.4)
Node plus bone	4 (13.3)
Visceral	1 (3.3)
Prostate bed	1 (3.3)
Biochemical	2 (6.7)

## Data Availability

Research data are not available at this time.
